# Electrical silencing of dendritic arborization neurons rescues toxic polyglutamine-induced locomotion defect

**DOI:** 10.1080/19336934.2025.2519687

**Published:** 2025-06-16

**Authors:** Hongyu Miao, Woo Jae Kim

**Affiliations:** HIT Center for Life Sciences (HCLS), School of Life Science and Technology, Harbin Institute of Technology, Harbin, China

**Keywords:** Polyglutamine (polyQ), neurodegenerative diseases, *Drosophila* melanogaster, dendritic arborization (da) neurons, locomotion, electrical silencing, potassium channels, neurodegeneration, therapeutic strategies, Huntington’s disease

## Abstract

This study investigates the effects of polyglutamine (polyQ) expansions on the locomotion of *Drosophila* larvae, focusing on the role of class IV dendritic arborization (da) neurons. PolyQ expansions are associated with neurodegenerative diseases like Huntington’s disease, and *Drosophila* is a valuable model organism for studying these diseases due to its genetic tractability and short generation time. We found that expressing a polyQ protein in class IV da neurons caused significant locomotion deficits. Specifically, larvae with polyQ expression exhibited slower crawling speed and increased turn frequency, indicating impaired movement. The most intriguing finding of our study was that electrically silencing class IV da neurons completely rescued the locomotion deficits caused by polyQ expression. By expressing a potassium channel that makes the neurons less active, we effectively reversed the locomotion defects. This suggests that modulating the activity of these neurons could be a promising therapeutic approach for treating polyQ diseases. Our findings have significant implications for understanding polyQ diseases and developing new therapeutic approaches. By electrically silencing these neurons, we may be preventing the harmful effects of polyQ-induced cation channels, which are thought to disrupt cellular function. This opens up exciting possibilities for exploring electrical silencing as a potential treatment for polyQ diseases, offering hope for future therapies that target the underlying mechanisms of these devastating conditions.

## Introduction

Polyglutamine (polyQ) diseases represent a family of neurodegenerative disorders characterized by the expansion of a polyQ tract within the protein coding sequence of affected genes. This expanded polyQ tract leads to the formation of toxic protein aggregates, which disrupt cellular functions and ultimately result in neuronal death [1]. The most common polyQ diseases include Huntington’s disease, spinobulbar muscular atrophy, and spinocerebellar ataxias. These disorders manifest with a wide range of symptoms, including movement abnormalities, cognitive decline, and psychiatric disturbances. Despite extensive research, the exact mechanisms underlying polyQ toxicity remain elusive, and effective treatments for these devastating diseases are still lacking [2,3].

*Drosophila melanogaster*, the fruit fly, has emerged as a powerful model organism for studying neurodegenerative diseases, including those caused by polyQ expansions. The simplicity of the fly nervous system, combined with its genetic tractability and short generation time, allows for rapid and efficient exploration of disease mechanisms and the screening of potential therapeutic interventions [4–6]. In *Drosophila* models of polyQ diseases, researchers commonly use transgenic techniques to express human polyQ-containing proteins, such as huntingtin or ataxin, in specific neurons [7]. These models recapitulate key features of human polyQ diseases, including the formation of intracellular aggregates, progressive neuronal loss, and behavioural phenotypes. By manipulating gene expression, studying protein localization, and examining cellular pathways in these models, researchers gain valuable insights into the pathogenic mechanisms of polyQ diseases. Additionally, the *Drosophila* model facilitates the identification of modifiers of polyQ toxicity, offering potential targets for therapeutic intervention [6,8,9].

*Drosophila melanogaster* larval dendritic arborization (da) neurons are a well-characterized subset of sensory neurons that play a crucial role in mediating the larval locomotion behaviour [10,11]. These neurons are organized into seven clusters along the ventral nerve cord and are responsible for detecting and responding to mechanical stimuli, such as touch and vibration, from the surrounding environment. These neurons consist of four distinct classes, which are repetitively organized along the body segments. Each class of da neurons possesses a unique and consistent dendritic branching pattern, likely tailored to their specific sensory functions. Notably, class III and class IV da neurons have dendrites that extensively cover the epidermis, providing a dense network for sensory input [12–16].

Research has shown that pathogenic polyQ proteins can lead to abnormalities in dendrites of da neurons, accompanied by changes in the actin cytoskeleton in *Drosophila* [17]. Additionally, Golgi outpost synthesis impaired by toxic polyglutamine proteins contributes to dendritic pathology in da neurons, and impaired dendritic morphology was associated to locomotion, and this phenotype could be restored by the genetic expression of Rac1 and CrebA [18]. The larval locomotion behaviour of *Drosophila melanogaster* serves as a sensitive and informative model for investigating the impact of polyQ expansions on neural circuits and motor function. These locomotor deficits are often associated with neuronal degeneration, altered neurotransmitter release, and disruptions in neural circuit function. The *Drosophila* larval locomotion model offers a powerful tool for dissecting the molecular and cellular mechanisms underlying polyQ toxicity and its impact on motor behaviour. Here, we report our finding that electrical silencing of class IV da neurons could completely rescue hypertoxic polyQ-induced impaired locomotion phenotype in *Drosophila* larvae [19]. These results would elucidate the potential cure of polyQ-mediated neurodegenerative disease.

## Results

### The reduction of dendritic complexity within class IV da neurons in Drosophila larvae gives rise to substantial changes in locomotor behaviors

**Figure 1.**
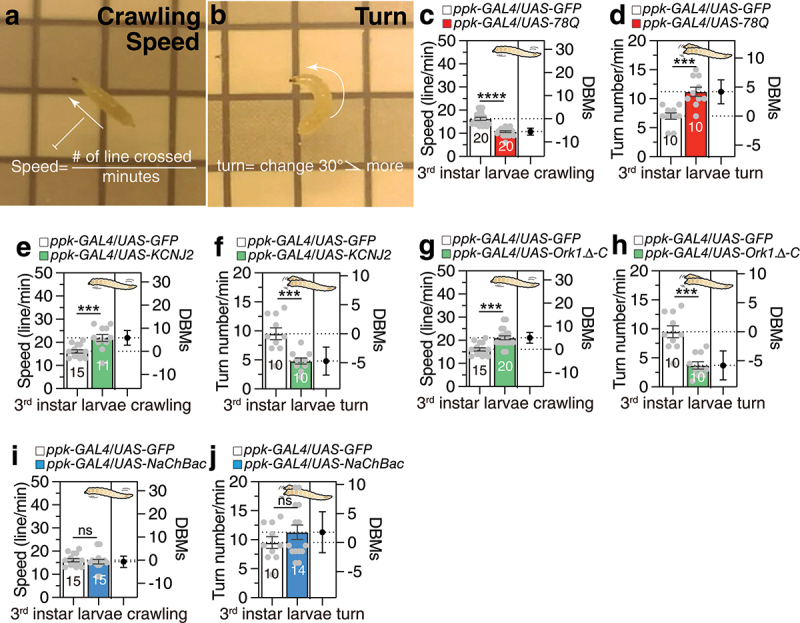
The dendritic complexity of class IV dendritic arborization (da) neurons in *Drosophila* larvae are closely linked to their locomotor behaviour. (a-b) Schematic representation of larval locomotion counting methods. For detailed methods, see the METHODS for a detailed description of the larval locomotion assay used in this study. (c-d) Locomotion assay of larvae that are expressing *UAS-MJD-78Q* (labelled as *UAS-78Q*) in da neurons via *ppk-GAL4*. (c) represents crawling speed, and (d) represents turn number within 1 min. White bars represent genetic controls that expressed GFP via *ppk-GAL4*. Dot plots represent the speed or turn number of each larvae. The mean value and standard error are labelled within the dot plot (black lines). Asterisks represent significant differences, as revealed by the Student’s t test, and ns represents non-significant differences (*p < 0.05, **p < 0.01, ***p < 0.001, ****p < 0.0001). (e-f) Locomotion assay of larvae for *ppk-GAL4* mediated electrical silencing via *KCNJ2*. (e) represents crawling speed, and (f) represents turn number within 1 min. (g-h) Locomotion assay of larvae for *ppk-GAL4* mediated electrical silencing via *Ork1.Δ-C*. (e) represents crawling speed, and (f) represents turn number within 1 min. (i-j) Locomotion assay of larvae for *ppk-GAL4* mediated electrical activating via *NaChBac*. (e) represents crawling speed, and (f) represents turn number within 1 min.

To explore the locomotor consequences of pathogenic polyQ expression in *Drosophila* larval da neurons, we adopted a straightforward approach involving video recording to quantify larval locomotion. Among the various locomotor behaviours, we selected crawling speed and turn frequency as two distinct yet interrelated measures of locomotion [20] (Figure 1a,b). Wild-type larvae were observed to crawl approximately 15 grid lines per minute and turn about 7 times per minute (white bars in Figure 1c,d). Among multiple expanded pathogenic polyQ transgenes, we selected spinocerebellar ataxia type 3 (SCA3; also known as Machado-Joseph disease, MJD-78Q) for analysis, as prior work demonstrated that *UAS-MJD-78Q* expression most severely disrupts the dendritic arborization of class IV neurons [19]. To investigate how polyQ expansion in class IV dendritic arborization (da) neurons affects locomotion, we expressed *UAS-MJD-78Q* (abbreviated as *UAS-78Q*) specifically in these neurons. Larvae expressing *UAS-78Q* exhibited significantly slower crawling speeds and more frequent turning behaviours compared to controls (Figure 1c,d). This demonstrates that restricted expression of expanded polyQ proteins in class IV da neurons is sufficient to disrupt locomotor behaviour. Employing *elav-GAL80* to selectively suppress GAL4 activity in neurons yielded divergent locomotor phenotypes in *ppk-GAL4/UAS-78Q* larvae [21], thus corroborating that the prior behavioural deficits were of neuronal origin (Figure S1a,b).

Prior research has indicated that when pathogenic polyQ proteins are expressed in adult *Drosophila* during the developmental process of neuronal dendrites, dendritic complexity is significantly diminished [17]. This reduction in dendritic complexity is linked to alterations in larval locomotion [18]. To validate that the reduction of dendritic complexity induced by MJD-78Q expression correlates with locomotor changes, we employed a distinct genetic approach capable of reducing dendritic complexity in class IV da neurons. Specifically, double-bromo and extraterminal (BET) domain proteins encoded by *fs(1)h* have been demonstrated to regulate dendritic complexity in these neurons, with their functional roles including both maintenance and potential modulation of arborization [22]. Remarkably, the targeted knockdown of *fs(1)h* in class IV da neurons recapitulated the locomotor phenotype observed with MJD-78Q expression (Figure S1c-d). These findings suggest that the reduction in dendritic complexity within class IV da neurons is associated with a decrease in crawling speed and an increase in turning frequency, indicative of locomotor behavioural changes in larvae.

### The electrical silencing of class IV da neurons results in contrasting locomotive defects compared to pathogenic polyQ expression

The successful silencing of multiple neuronal types in the *Drosophila* system through the expression of inwardly rectifying potassium channels, such as human KCNJ2/Kir2.1, has been demonstrated [23]. It has been reported that acute overexpression of KCNJ2 using SN-GAL4 to silence all sensory neurons at the third larval instar results in the immobilization of larvae after a 30-min exposure to restrictive temperature [10]. Although KCNJ2 has been expressed in class IV da neurons in adult flies [24–26], we found no reports of KCNJ2 expression specifically in *ppk-GAL4*-expressing class IV da neurons at the third instar larval stage. Surprisingly, the expression of KCNJ2 only in class IV da neurons increased larval crawling speed but decreased turning frequency (Figure 1e,f). Similarly, the expression of the *Drosophila* inward rectifier potassium channel Ork1.Δ-C also elicited a comparable locomotion phenotype (Figure 1g,h).

By overexpressing these potassium channels, the neuronal membrane potential is shifted to a less excitable state, inhibiting action potential generation and reducing overall neuronal activity. This is achieved by altering the resting membrane potential and the threshold for action potential initiation [23]. In contrast, the expression of tetanus toxin light chain (TNT) specifically inhibits vesicle-associated membrane protein (VAMP) 2 and 3, which are essential for synaptic vesicle fusion. By inhibiting this process, TNT blocks the release of neurotransmitters from presynaptic terminals, thereby preventing synaptic transmission [27,28]. When we expressed UAS-TNT in class IV da neurons, crawling speed was dramatically decreased and turn frequency significantly increased (Figure S1e-f). This data suggests that electrical silencing of class IV da neurons elicits a completely different effect compared to the inhibition of synaptic transmission. Furthermore, the expression of TNT shows a phenocopy of the expression of MJD-78Q, raising the possibility that electrical silencing might act through a mechanism distinct from synaptic transmission inhibition in these neurons.

To investigate whether the electrical modification of class IV da neurons is associated with the locomotion changes of larval behaviour, we decided to electrically activate the neurons by expressing the bacterial sodium channel NaChBac. It has been reported that the genetic expression of NaChBac causes hyper-excitation of many types of neurons in *Drosophila* [29,30]. Interestingly, the expression of NaChBac in class IV da neurons had no significant effect on larval locomotion (Figure 1i,j), suggesting that electrical silencing, not electrical stimulation of class IV da neurons, is associated with larval locomotion behaviour.

### Electrical silencing of class IV da neurons specifically rescues polyQ-induced locomotion defects

Our results indicate that electrical silencing of class IV da neurons specifically modulates larval locomotion behaviour in the opposite direction to the polyQ-induced phenotype. Thus, we hypothesized that electrical silencing with polyQ expression might rescue polyQ-induced locomotive defects.

**Figure 2.**
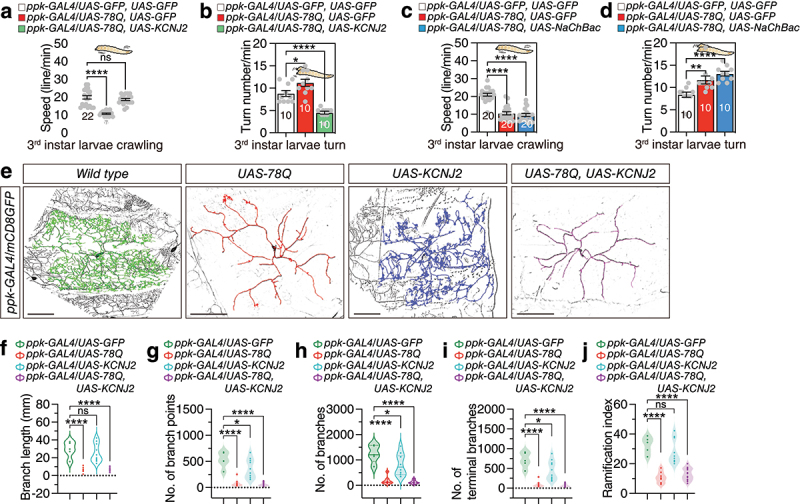
Electrical inhibition of class IV dendritic arborization (da) neurons effectively mitigates locomotor impairments induced by polyQ protein expression. (a-b) Genetic rescue experiments of locomotion assay for *ppk-GAL4* mediated expression of *MJD-78Q* via *KCNJ2* resulted in electrical silencing. (a) represents crawling speed, and (b) represents turn number within 1 min. (c-d) Genetic rescue experiments of locomotion assay for *ppk-GAL4* mediated expression of *MJD-78Q* via *NaChBac* resulted in electrical activating. (c) represents crawling speed, and (d) represents turn number within 1 min. (e) The morphological alterations in class IV da neurons overexpressing pathogenic polyQ proteins or KCNJ2 were examined. The genotypes are represented from left to right as indicated in the accompanying figure. Scale bars represent 100 µm. (f-j) Analysis and quantification of the morphological alterations in class IV da neurons overexpressing polyQ proteins or KCNJ2. The sample sizes (n) from left to right are as follows: control group *n* = 5, polyQ overexpression group *n* = 7, KCNJ2 overexpression group *n* = 10, and the group with co-overexpression of polyQ and KCNJ2 *n* = 14. (f) represents total branch length, (g) represents the number of branch points, (h) represents the number of branches, (i) represents the number of terminal branches and (j) represents ramification index. For detailed methods, see the METHODS for a detailed description of the quantitative analysis of dendritic morphology.

As we hypothesized, coexpression of KCNJ2 with MJD-78Q could rescue polyQ-induced reduced crawling speed (Figure 2a) and increased turn frequency (Figure 2b). Coexpression of Ork1.Δ-C with MJD-78Q also rescued polyQ-induced locomotive defects (Figure S2a,b), suggesting that electrical silencing of class IV da neurons could rescue the pathogenic effects of polyQ.

Neither electrical stimulation by NaChBac expression nor inhibition of synaptic transmission by TNT expression could alter the polyQ-induced locomotive defects (Figure 2c,d and Figure S2c,d), suggesting that these transgene expressions in class IV da neurons could not rescue polyQ-induced locomotive defects. Given the age-dependent progression of polyQ disorders [31], we extended our analysis to adult flies to assess whether KCNJ2 rescues locomotor deficits in aged animals. Consistent with larval phenotypes, *ppk-GAL4*-driven expression of *UAS-78Q* in class IV da neurons resulted in significant climbing defects and reduced activity in 30-day-old adult flies (Figure S2e-g). Strikingly, co-expression of *UAS-KCNJ2* with UAS-78Q restored the most of locomotive behaviours to near wild-type levels (Figure S2h, i). These results demonstrate that KCNJ2 mitigates polyQ-induced locomotor deficits not only in larvae but also in aged adults, underscoring its conserved role in counteracting neurotoxic effects across developmental stages. Meanwhile, although the expression of 78Q in the trachea also leads to a decrease in larval claw speed, KCNJ2 does not rescue this decrease in the trachea (Figure S2k-l).

To assess whether KCNJ2 rescues polyQ-induced structural deficits in class IV da neurons, we performed live imaging of larval da neurons using mCD8GFP (Figure 2e). Expression of *UAS-78Q* in class IV neurons (*ppk-GAL4>UAS-78Q*) caused severe dendritic arborization defects, including reduced branch complexity and terminal retraction (Figure 2f, quantified in 2 g-j). Strikingly, co-expression of *UAS-KCNJ2* with *UAS-78Q* (ppk-*GAL4>UAS-78Q; UAS-KCNJ2*) failed to restore dendritic complexity (Figure 2e-j), as quantified by Sholl analysis (Figure S2j) and terminal branch counts (Figure 2f-j). These findings demonstrate that while KCNJ2 rescues locomotor behaviour (Figure 1c,d; Figure S2e-i), it does not mitigate polyQ-induced dendritic structural damage, suggesting its functional rescue operates independently of morphological restoration.

## Discussion

Polyglutamine expansions cause devastating neurodegenerative diseases like Huntington’s disease and spinocerebellar ataxias, characterized by toxic protein aggregates and neuronal death. Despite extensive research, the mechanisms behind polyQ toxicity remain elusive, and effective treatments are lacking. We used the *Drosophila melanogaster* model to investigate the role of class IV da neurons in polyQ-induced locomotor deficits. We found that expressing a truncated mammalian MJD1 protein with an expanded glutamine repeat (MJD-78Q) in class IV da neurons significantly reduced crawling speed and increased turn frequency, indicating locomotor impairment. This phenotype was recapitulated by reducing dendritic complexity in class IV da neurons, confirming the importance of dendritic complexity of class IV neurons in locomotion. Surprisingly, electrical silencing of class IV da neurons with inwardly rectifying potassium channels (KCNJ2 and Ork1.Δ-C) completely rescued the locomotor defects induced by MJD-78Q expression. This rescue was specific to electrical silencing, as neither electrical activation nor inhibition of synaptic transmission with other transgenes had the same effect. These findings suggest that modulating the activity of class IV da neurons holds promise as a potential therapeutic approach for treating polyQ-mediated neurodegenerative diseases.

The *Drosophila* larval body wall is innervated by 42 sensory neurons per hemisegment, arranged in a modality-specific pattern. While motor patterns can be generated independently of sensory input, peristaltic muscle contraction waves and locomotion are impaired in the absence of sensory input, resulting in slower and less coordinated movement. Dendritic arborization (da) neurons are a well-characterized subset of sensory neurons, and their role in locomotion has been extensively studied [23]. Previous research demonstrated that expressing polyQ in all da neurons reduced turning frequency without affecting crawling speed [18]. This discrepancy may be attributed to our targeted expression experiments, which restricted polyQ expression to class IV da neurons. Future studies comparing polyQ toxicity across da neuron classes (e.g. class I proprioceptors vs. class IV nociceptors) could elucidate whether dendritic degeneration or ion channel dysregulation exhibits neuron-type specificity, potentially informing therapeutic strategies for distinct neurodegenerative contexts.

Our findings raise questions about the specificity of polyQ toxicity for potassium channels. Potassium channels such as KCNJ2 and Ork1, unlike sodium channels, possess cytoplasmic domains rich in charged amino acids [32] that may interact preferentially with polyQ expansions via electrostatic or hydrophobic forces. While sodium channels were not explicitly tested here, their relative resistance to polyQ interference may reflect differences in subcellular localization or molecular binding affinity.

Notably, potassium channel dysfunction in polyQ diseases is not limited to *Drosophila*. For example, in a mouse model of the huntingtin gene (Q175), reduced dendritic excitability can be attributed to enhanced opening of Kv4 K^+^ channels [33].

Despite significant research efforts, effective treatments for polyQ diseases remain elusive. Challenges include the complexity of disease mechanisms, the difficulty of targeting protein aggregates, and the lack of reliable animal models. Therapeutic strategies for polyQ diseases encompass a diverse range of approaches targeting various aspects of the disease pathology. These include enhancing protein degradation through autophagy or the ubiquitin-proteasome system, reducing protein expression with RNA interference or antisense oligonucleotides, and developing small molecules to modulate specific pathways involved in polyQ toxicity. Additionally, strategies aim to prevent protein misfolding and aggregation through chaperone overexpression and to protect neurons with neuroprotective compounds. Stem cell-based therapies, gene editing technologies, symptomatic treatments, and combination therapies also hold promise. Precision medicine approaches aim to tailor treatments to individual patients, optimizing therapeutic outcomes [34,35].

Our findings reveal a critical dissociation between functional and structural rescue in polyQ toxicity: while KCNJ2 restores locomotor behaviour in *ppk-GAL4>UAS-78Q* larvae and adults, it does not reverse dendritic arborization defects (Figure 2e-j). This suggests that KCNJ2 ameliorates locomotor deficits by targeting electrophysiological dysfunction – such as restoring ion homoeostasis or stabilizing synaptic transmission – rather than mitigating structural damage. A similar uncoupling of functional and morphological recovery has been observed in Drosophila models of mechanosensation, where modulation of TRP channels rescues nociceptive behaviour without restoring dendritic complexity [36]. This aligns with broader evidence that ion dyshomeostasis, particularly calcium overload, is a central driver of polyQ-induced neuronal dysfunction [37]. For example, expanded polyQ tracts in Huntington’s disease models form aberrant cation channels that depolarize membranes and disrupt calcium signalling, leading to excitotoxicity [38]. By counteracting depolarization through its inwardly rectifying K^+^ conductance, KCNJ2 may rebalance membrane potential and calcium flux, thereby rescuing locomotor circuits downstream of class IV neuron dysfunction. These findings underscore the therapeutic potential of targeting electrophysiological pathways to alleviate functional deficits in neurodegenerative disorders, even in the absence of structural repair.

Electrical modification, specifically deep brain stimulation (DBS), has been explored as a potential therapeutic approach for various neurodegenerative diseases, with Alzheimer’s disease (AD) being one of the most extensively investigated [39]. Recent research has generated compelling evidence supporting the ‘toxic-channel hypothesis’ as a novel conceptual framework for elucidating the pathogenic mechanisms underlying polyQ diseases [34]. The hypothesis suggests that expanded polyglutamine tracts within disease-causing proteins can form cation channels, disrupting cellular ion homoeostasis and leading to cellular dysfunction and death. The toxic-channel hypothesis proposes that aggregate formation may be a secondary response or a protective mechanism, rather than the primary cause of toxicity [38,40–42]. This hypothesis opens up new avenues for therapeutic intervention, potentially involving the modulation of polyQ channel activity. While direct translation to human therapies remains speculative, our findings parallel advances in neuromodulation, such as optogenetic vision restoration [43], where functional recovery is achieved despite irreversible structural damage. This underscores the potential of electrical silencing strategies to alleviate circuit-level dysfunction in neurodegenerative contexts, even in the absence of structural repair.

Our results suggest that electrical silencing of class IV da neurons can rescue the locomotive defects induced by polyQ expression, potentially by inhibiting the formation or activity of polyQ-induced cation channels. This hypothesis is consistent with the ‘toxic-channel hypothesis’, which posits that expanded polyglutamine tracts can form ion channels that disrupt cellular function. By electrically silencing class IV da neurons, we may be preventing the toxic effects of these polyQ-induced cation channels, thereby restoring normal locomotion behaviour. Current treatment approaches for polyQ diseases primarily aim to alleviate symptoms, slow disease progression, and enhance patient quality of life. Our findings indicate that electrical silencing may offer an alternative avenue for treating polyQ diseases by inhibiting the formation or activity of polyQ-induced cation channels, which are hypothesized to contribute to disease pathogenesis. This suggests that electrical silencing could be a novel therapeutic strategy to explore for the potential cure of polyQ diseases.

While we did not directly measure *fs(1)h* or KCNJ2 expression levels in this study, prior literature suggests that polyQ proteins like MJD-78Q may disrupt transcriptional or translational machinery broadly [34,44,45]. It is plausible that MJD-78Q could indirectly influence fs(1)h/KCNJ2 activity via epigenetic or post-translational mechanisms, though this remains to be validated. Future studies exploring these molecular interactions would further clarify the regulatory network underlying dendritic defects in our model.

## Methods

### Fly strains and rearing

*Drosophila melanogaster* was raised on cornmeal-yeast medium at similar densities to yield larvae with similar body sizes. Flies were kept in 12 h light: 12 h dark cycles (LD) at 25°C (ZT 0 is the beginning of the light phase, ZT12 beginning of the dark phase). To reduce the variation from genetic background, all flies were backcrossed for at least three generations to *Canton-S* strain.

The following lines were obtained from Bloomington Stock Center: *ppk-GAL4* (32079), *UAS-GFP* (6658), *UAS-KCNJ2* (6595), *UAS-Ork1.Δ-C* (8928), *ppk-GAL4, UAS-mCD8GFP* (8749), *btl-GAL4, UAS-Act5C-GFP* (8807);* UAS-NaChBac* (9469), *UAS-TNT* (28838).

The following lines were obtained from Vienna Drosophila Resource Center: *UAS-fs(1)h-RNAi* (51227).

The following line was obtained from Dr. Nancy. M. Bonini (University of Pennsylvania, Philadelphia): *UAS-MJD-78Q*.

### Larvae locomotion assay

Our larvae locomotion analysis is based on previous methods [46]. To document the locomotor behaviours of larvae, a 10-cm-diameter silicone plate was positioned on a cool-operating, evenly light panel situated beneath a smartphone (OPPO, A72) at 29.97 frames per second (fps). Transparent silicone plates with a grid of 2 mm sides were drawn on the back to count larval locomotion. The entire procedure was carried out at room temperature (25°C) and a humidity level of 50%. A single 3rd instar larva of the wandering stage was removed from the wall of a food vial, washed briefly with distilled water, then transferred by brush to the centre of a silicone plate. Before video recording, larvae were allowed 30 s to recover from handling and to adapt to the silicone plate and the illumination. Each larva was recorded for 1 min of video, and the locomotion was manually counted after all animals had been recorded. ‘Crawling Speed’ was calculated as: number of line crossed/minutes, each rotation of the larval head at an angle greater than 30 degrees is recorded as a ‘Turn’. Line crossings per minute were used as a validated proxy for crawling speed, as described in prior studies [46]. For reference, 1 line crossing ≈2 mm (plate grid spacing), enabling approximate conversion to mm/min where applicable.

### Adult locomotion assay

To image fly locomotion, a custom-LED array was placed under the stage to serve as backlight. A 2-mm transparent acrylic board sheet was placed on top of the LED array to produce homogeneous illumination. Fly behaviours were recorded using a camera with 1920 × 1080 30 hz (1080p30). Behavioral arenas were custom-built from opaque transparent acrylic board sheets. Chambers were 30 mm in diameter and 2 mm in height. Each fly group was aspirated into a separate chamber and placed on the stage. Flies were observed for 30 min to ensure that no gross motor defects were present before video acquisition was initiated.

Image segmentation was performed using the custom software Fly Trajectory Dynamics Tracking (FlyTrDT) in python [47]; First, FlyTrDT identifies and quantifies fruit flies into elliptical pixels, and we extracted 2 main features from the videos: 1) The average forward speed and 2) The trajectory map of the fly group in each chamber. For the average forward speed of each group, FlyTrDT records the moving speed of each indoor fruit fly and analyzes the average speed of 10 fruit flies per second. For the trajectory map, FlyTrDT records the movement of each indoor fruit fly, then analyzes and plots the movement trajectories of 10 fruit flies in each chamber, including the movement trajectories of the entire group and the movement trajectories of each fruit fly in the group.

### Quantitative analysis of dendritic morphology

Male third-instar larvae were meticulously excised and subjected to a thorough washing procedure with PBS (Phosphate buffered saline). Subsequently, these larvae were anesthetized using ice and promptly underwent imaging to capture the body surface sensory neurons with the ECHO Revolve microscope (ECHO, San Diego, CA). Owing to the diminished fluorescence signal associated with the sensory neurons of specific genotypes, we utilized the ‘Trainable Weka Segmentation [48]’ plugin, which is grounded in machine learning algorithms, integrated within the ImageJ software suite. This plugin was employed to process the original images and to facilitate their binarization. Following this preprocessing step, we engaged the SNT (Simple Neurite Tracer [49]) plugin available in ImageJ to delineate the dendritic arbours, allowing for the quantification of key morphological parameters, including the enumeration of dendritic branch terminals and the cumulative length of the dendritic branching network. For the purpose of Sholl analysis, we applied this tracing methodology to assess the dendritic branching pattern at 1 μm intervals along the circumscribed concentric circles encompassing the dendritic trees.

### Statistical tests

As the locomotion data of larvae showed normal distribution (Kolmogorov–Smirnov tests, *p* > 0.05), we used two-sided Student’s t tests. Analysis of variance (ANOVA) was conducted for all experimental conditions and Tukey’s test to determine significance within groups examined. Each figure shows the mean ±standard error (s.e.m) (**** = p < 0.0001, *** = p < 0.001, ** = p < 0.01, * = p < 0.05). All the analysis was done in GraphPad (Prism9). Besides traditional t-test for statistical analysis, we added estimation statistics for all two group comparing graphs. In short, ‘estimation statistics’ is a simple framework that – while avoiding the pitfalls of significance testing – uses familiar statistical concepts: means, mean differences, and error bars. More importantly, it focuses on the effect size of one’s experiment/intervention, as opposed to significance testing [50]. In comparison to typical NHST plots, estimation graphics have the following five significant advantages such as (1) avoid false dichotomy, (2) display all observed values, (3) visualize estimate precision, (4) show mean difference distribution. And most importantly (5) by focusing attention on an effect size, the difference diagram encourages quantitative reasoning about the system under study [51]. In 2019, the Society for Neuroscience journal eNeuro instituted a policy recommending the use of estimation graphics as the preferred method for data presentation [52].

## Supplementary Material

Supplemental Material
