# Supplementary figures and images for: Electrical silencing of dendritic arborization neurons rescues toxic polyglutamine-induced locomotion defect
Source: Fly (Austin). 2025 Jun 16;19(1):2519687. doi: 10.1080/19336934.2025.2519687 (PMC12184177; doi:10.1080/19336934.2025.2519687)

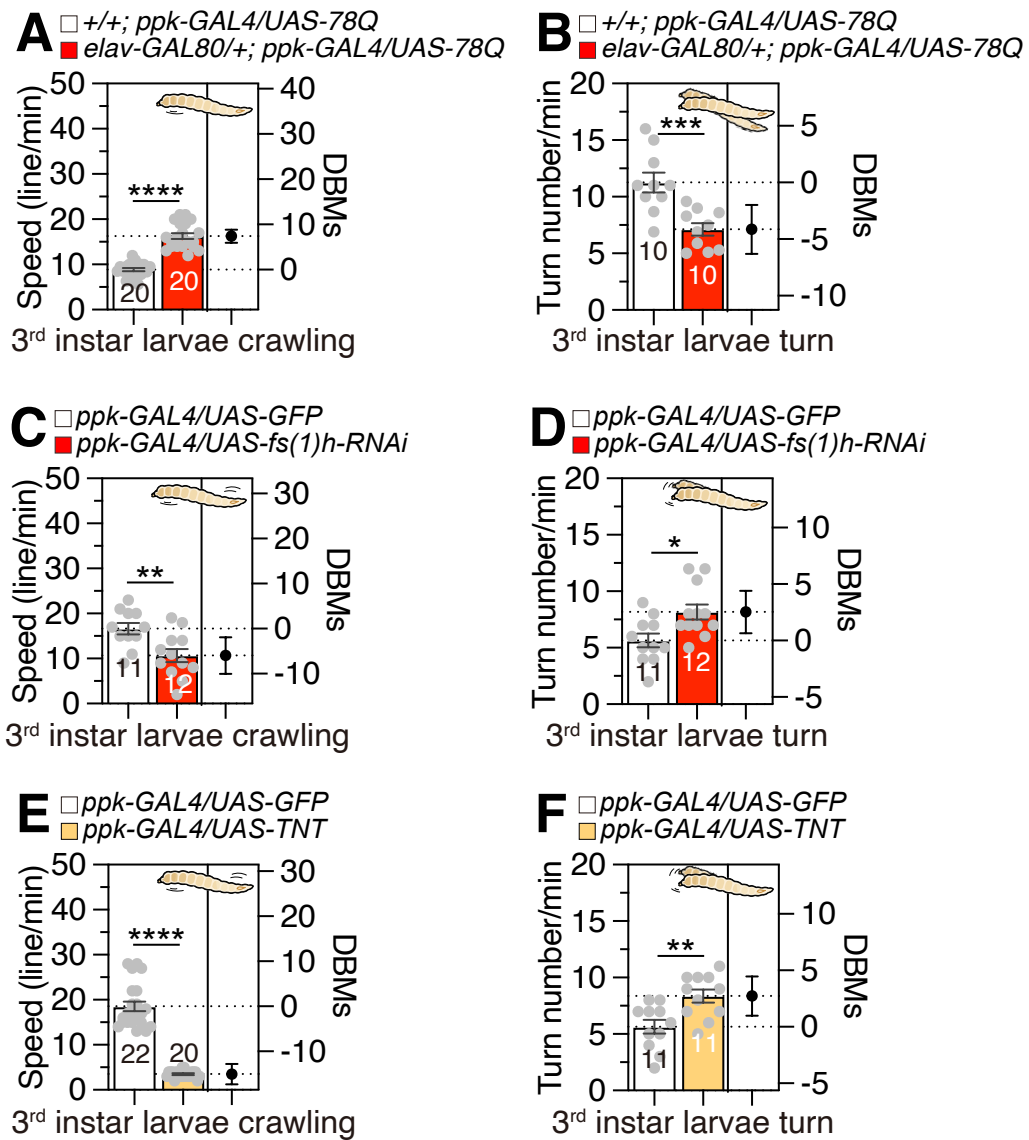

***PolyQ, Fig.S1***

Supplement: Supplemental Material [file KFLY_A_2519687_SM4356.zip › 2025_PolyQ_FigS1.pdf]

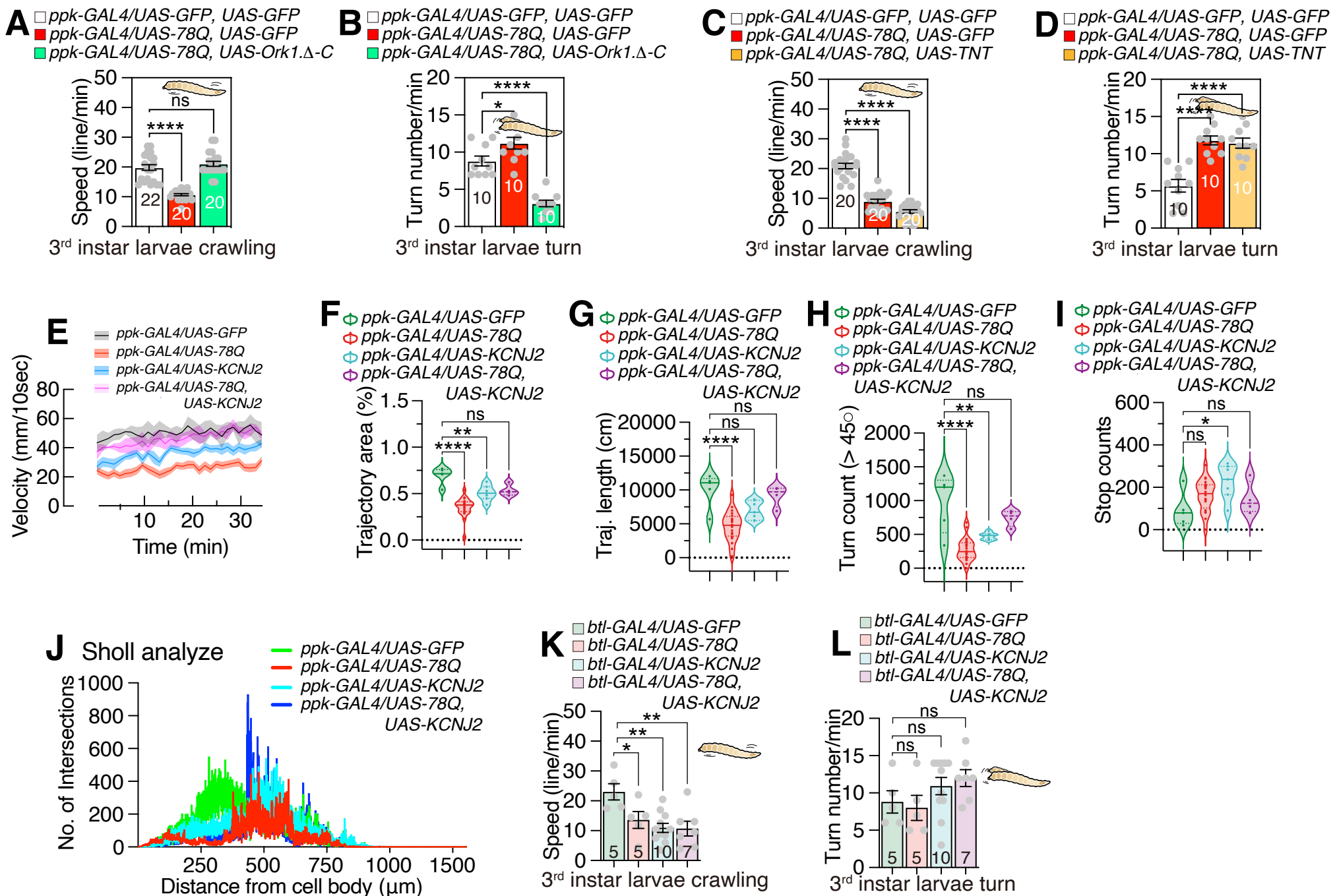

Supplement: Supplemental Material [file KFLY_A_2519687_SM4356.zip › 2025_PolyQ_FigS2.pdf]
